# Antifibrotic Effect of Combination of Nilotinib and Stem Cell-Conditioned Media on CCl_4_-Induced Liver Fibrosis

**DOI:** 10.1155/2020/6574010

**Published:** 2020-02-03

**Authors:** Gamal Shiha, Ahmed Nabil, Ahmed Lotfy, Reham Soliman, Ayman A. Hassan, Islam S. Ali, Doaa F. Gad, Faten Zahran

**Affiliations:** ^1^Egyptian Liver Research Institute and Hospital (ELRIAH), Mansoura, Egypt; ^2^Hepatology and Gastroenterology Unit, Internal Medicine Department, Faculty of Medicine, Mansoura University, Mansoura, Egypt; ^3^Biotechnology and Life Sciences Department, Faculty of Postgraduate Studies for Advanced Sciences (PSAS), Beni-Suef University, Egypt; ^4^Tropical Medicine Department, Faculty of Medicine, Port Said University, Port Said, Egypt; ^5^Delta University for Science and Technology, Egypt; ^6^Biochemistry Department, Faculty of Science, Zagazig University, Egypt

## Abstract

Liver fibrosis is the excessive extracellular matrix accumulation of proteins, such as collagen, which follows the chronic liver diseases. Advanced liver fibrosis leads to cirrhosis and liver failure. Nilotinib is a second-generation tyrosine kinase inhibitor, which showed antifibrotic efficacy. Stem cell therapy still has some limitations such as oncogenesis, unexpected differentiation, and ethical consideration. Stem cells secrete cytokines and growth factors that showed paracrine-mediated antifibrotic and anti-inflammatory effects in vivo and in vitro. Thus, stem cell-conditioned medium (SC-CM), which contains the secretory proteins of stem cells, may have an antifibrotic role. This study was carried out to examine the antifibrotic effect of Nilotinib and stem cell exosomes on CCl_4_-induced liver fibrosis in rats. Male Wistar rats were injected intraperitoneally with CCl_4_ twice a week for 9 weeks and given daily treatments of Nilotinib (20 mg/kg), stem cell exosomes (0.5 ml/rat), and the combination treatment of Nilotinib and stem cell exosomes during the last 5 weeks of CCl_4_ intoxication. Liver fibrosis and also antifibrotic efficacy of the treatments were estimated with liver function tests, oxidative stress parameters, apoptotic parameters, histopathological examination, and hydroxyproline contents. Results showed that the combination of Nilotinib and stem cell-conditioned media had more antifibrotic effects than each one alone (*P* value < 0.001).

## 1. Introduction

Fibrosis is a common pathological process for the majority of liver diseases which leads to liver cirrhosis and/or hepatocellular carcinoma. It is a consequence of almost all chronic liver diseases predominantly arising from viral, alcohol-induced, autoimmune, and metabolic etiologies [1]. Fibrosis results from unregulated wound healing and is characterized by the progressive replacement of functional hepatic tissue with highly cross-linked collagen I/III-rich extracellular matrix; it disrupts both the normal architecture and functions of the liver especially in the end stage of cirrhosis. Fibrosis is also considered a precancerous state that provides microenvironments in which primary tumors may develop [2].

Tyrosine kinase activation has been involved in fibrogenesis. Tyrosine kinases are implicated in various cellular activities, including differentiation, apoptosis, metabolism, and growth [3]. The phosphorylated tyrosine residues are the common mode of action of these enzymes using ATP. There are 2 classes of tyrosine kinases: receptor tyrosine kinases, like the PDGF receptors, and nonreceptor tyrosine kinases, like the Abelson kinase (c-Abl). Besides the tyrosine kinases' physiological roles, recent studies have shown their activation role in carcinogenesis pathophysiology, fibrogenesis, rheumatoid arthritis, and vascular remodeling. So, inhibitors that block tyrosine kinase activity may be helpful for the treatment of these diseases [4].

The introduction of tyrosine kinase inhibitor therapy, in the form of Imatinib (1^st^ generation TKIs), has significantly improved the outcome of patients with chronic myeloid leukemia (CML). Nilotinib belongs to the second-generation TKIs. It was designed to overcome the resistance of Imatinib in chronic myelogenous leukemia (CML) [5].

Several studies showed that Nilotinib can control hepatic fibrosis by regulating levels of proinflammatory cytokines, primarily interleukin- (IL-) 1 and IL-6 [6–9]. In an earlier study, we compared Nilotinib, Imatinib, and silymarin in their effect as antifibrotic agents [4]; we found that Nilotinib is better than silymarin and less toxic than Imatinib, and also, we found that Nilotinib induces apoptosis and autophagic cell death of activated hepatic stellate cells via inhibition of histone deacetylases [7]. We also studied the therapeutic effect of stem cells in liver fibrosis and found that they are comparable to Nilotinib as an antifibrotic agent [8]. Stem cell therapy applications still have many obstacles such as oncogenicity; it may exert unexpected differentiation, in addition to ethical consideration [10].

Stem cells release several products in a paracrine fashion like extracellular vesicles (EVs) in conditioned medium [10]. Extracellular vesicles which are secreted by cells are generally defined as microvesicles, cell-derived vesicles, microparticles, shedding vesicles, and exosomes [10]. Exosomes are lipid vesicles which contain evolutionarily conserved sets of proteins including tetraspanins (CD81, CD63, and CD9), heat shock proteins (HSP60, HSP70, and HSP90), and tumor susceptibility gene 101 and have been reported to have multiple functions including angiogenesis, cell proliferation, and collagen reduction [11].

Several studies found that mesenchymal stem cell-conditioned medium (MSC-CM) has a therapeutic effect in liver fibrosis [12, 13]. Moreover, some clinical trials are in progress to assess MSC-CM therapeutic potential and to determine the optimal dose, the appropriate time for the administration of exosomes, and the administration route that accomplishes maximal efficacy without causing adverse effects [14, 15]. The aim of this work is to study the antifibrotic effect of Nilotinib and MSC-CM combination on CCl_4_-induced liver fibrosis in rats as compared to each one alone.

## 2. Materials and Methods

### 2.1. Experimental Animals

Sixty male Wistar rats, weighing 180-200 gm, were used in this study. Rats were obtained from the Medical Experimental Research Center (MERC), Faculty of Medicine, Mansoura University, Egypt. They were housed in Medical Experimental Research Center (MERC), at 8-10 weeks of age in a controlled environment at 21 ± 2°C, 50 ± 5% relative humidity, and a 12 h light/dark cycle. Rats were kept for one week prior to the experiment for adaptation to the new environment. Rats were kept in cages (5 rats per 1 cage) at constant environment and nutritional condition throughout the period of the experiment. All rats received humane care according to the National Institutes of Health criteria for care of laboratory animals. All the animal experiments were conducted at MERC by veterinary doctors in summer 2018.

### 2.2. Study Design

The experimental animals were assigned to groups by using Statistical Package of Social Science (SPSS) program for Windows (Standard version 21). Rats were divided randomly into 6 main groups (10 each) as follows. Group I (healthy control): rats received 1 ml/kg corn oil twice a week for 9 weeks. Group II (CCl_4_ group): rats received 1 ml/kg of 50% (*v*/*v*) CCl_4_ solution in olive oil twice a week for 9 weeks [16]. Group III (free media group): rats received 1 ml/kg of 50% (*v*/*v*) CCl_4_ solution in olive oil+exosome free media (fetal bovine serum) (injected in the rats' tail vein at a dose of 0.5 ml/rat/day) during the last 5 weeks of CCl_4_ intoxication. Group IV (CCl_4_+Nilotinib): rats received 1 ml/kg of 50% (*v*/*v*) CCl_4_ solution in olive oil+Nilotinib (formerly AMN107; Tasigna®) which was generously supplied by Novartis (Basel, Switzerland). The Nilotinib dose is 20 mg/kg/day, 1% *w*/*v* in saline, 1 ml/kg by gavage during the last 5 weeks of CCl_4_ intoxication [4]. Group V (CCl_4_+stem cell exosomes): rats received 1 ml/kg of 50% (*v*/*v*) CCl_4_ solution in olive oil+stem cell exosomes (injected in the rats' tail vein at a dose of 0.5 ml/rat/day) during the last 5 weeks of CCl_4_ intoxication [17]. Group VI (CCl_4_+stem cell exosomes+Nilotinib): rats received 1 ml/kg of 50% (*v*/*v*) CCl_4_ solution in olive oil+stem cell exosomes (injected in the rats' tail vein at a dose of 0.5 ml/rat/day)+Nilotinib (20 mg/kg/day, 1% *w*/*v* in saline, 1 ml/kg by gavage) during the last 5 weeks of CCl_4_ intoxication.

Rats from each group were sacrificed under general anesthesia with thiopental. A laparotomy and hepatectomy were performed. Blood samples were withdrawn by cardiac puncture and centrifuged at 2000 g for 10 min for serum preparation. The liver samples were cut into halves. One half is stored in formaldehyde for the histopathological examination, and the other half is stored in liquid nitrogen for the other tests.

### 2.3. Preparation of Stem Cell-Conditioned Media

Liver mesenchymal stem cells isolated from 2 g of the liver from Wistar rat were extensively washed in PBS. Liver piece was minced and incubated in alpha-MEM supplemented with collagenase (2 mg/ml) at 37°C for 60 min and trypsinized (10 mg/ml) for 45 min. After the digestion was complete, the sample was suspended in 10 ml alpha-MEM and cells were recovered by centrifugation for 10 min at 500 g. This step was repeated twice. The cell pellet was suspended in 10 ml of alpha-MEM and centrifuged again to remove blood remains. The cell pellet was suspended in 10 ml of DMEM+10% FBS+1% antibiotic-antimycotic solution (Thermo Scientific, USA) and maintained for selection and expansion in a Petri dish in a 95% air, 5% CO_2_ humidified atmosphere at 37°C.

After 3 passages, they were seeded at 10000 cells/cm^2^ and incubated in a complete culture medium for one day. The liver mesenchymal stem cells were washed 3 times with phosphate buffer saline (PBS) and incubated in serum-free basal medium (Lonza) for 48 h. Then, the supernatant was collected for centrifugation, filtration, and concentration at 1500 × g for 10 min [18].

### 2.4. Evaluation of Liver Functions

Albumin levels and serum alanine aminotransferase (ALT) were estimated by using commercial kits from Biomed Diagnostics.

### 2.5. Hepatic Histopathological Evaluation

Histopathology examinations were performed on animals from each experimental group. To determine the amount of fibrotic tissue in the liver, liver tissue was collected after 9 weeks of CCl_4_ induction. Collected liver tissue was fixed, embedded, cut into 10 *μ*m thick sections, and stained with standard hematoxylin and eosin staining. Then, slides were stained with Masson trichrome as routine connective tissue stain. Images were captured from each section randomly, and a quantitative analysis of fibrotic area was performed using the ImageJ software (version 1.6.0_20, National Institutes of Health, Bethesda, MD) as previously reported [19].

### 2.6. Measurement of Hepatic 4-Hydroxyproline Content

Liver specimens (50 mg) were hydrolyzed, and the supernatant was added to chloramine T solution, then incubated, followed by the addition of Ehrlich's solution. The final mixture was incubated, and the optical density was estimated at 560 nm. The value of 4-hydroxyproline was expressed as *μ*g/g wet tissue [20].

### 2.7. Hepatic Oxidative Stress Parameters

#### 2.7.1. Measurement of Hepatic Malondialdehyde

1-Methyl-2-phenyl-indole was added to a sample of liver homogenate. After adding hydrochloric acid, samples were mixed well. The samples were incubated at 45°C. Then, samples were cooled and centrifuged, and the optical density was estimated at 586 nm [21].

#### 2.7.2. Measurement of Hepatic Nitric Oxide Content

Liver homogenate was added to sodium hydroxide, and zinc sulphate was added for deproteinization. This mixture was centrifuged, and the resultant supernatant was added to VCl_3_ in HCl and Griess reagent. After incubation, samples were read at 540 nm. Concentration of nitric oxide was measured from a NaNO_3_ standard curve [22].

#### 2.7.3. Measurement of Hepatic Superoxide Dismutase Activity

The liver homogenate was mixed with Tris-HCl, then pyrogallol was added. The change in the optical density per minute was estimated for the samples by monitoring the rise in the optical density at 420 nm. The inhibition percentage for the samples was calculated by running a blank tube under the same conditions [23].

#### 2.7.4. Measurement of Hepatic Catalase Activity

The liver homogenate was incubated with hydrogen peroxide and methanol. The enzymatic reaction was initiated by the addition of a catalase-containing sample. The reaction mixture was incubated with continuous shaking for 20 minutes. The enzymatic reaction was terminated by the addition of KOH solution. The absorbance was measured at 550 nm [24].

#### 2.7.5. Measurement of Hepatic Nitric Oxide Synthase Activity

The liver homogenate was added to the reaction mixture containing hydroxyethyl piperazine ethane sulfonic acid (HEPES), NADPH L-arginine, and was then incubated. The activity of nitric oxide synthase was calculated from the difference between samples and blank reading [25].

#### 2.7.6. Measurement of Hepatic Glutathione Reductase Activity

Liver homogenate and GSSG were added in a spectrophotometer cuvette. NADPH was added and the absorbance was recorded at 340 nm. The sample net rate was estimated by subtracting the rate for the blank [26].

### 2.8. Hepatic Apoptotic Markers

#### 2.8.1. Annexin V Staining Assay

A suspension of liver cells was incubated, and the supernatant was collected and the adherent cells were trypsinized. The collected cells were washed with PBS and centrifuged. The pellet was resuspended in PBS. 400 *μ*l of cells+incubation buffer were added with Annexin. The cells were analyzed with flow cytometry without washing the cells [27].

#### 2.8.2. Determination Percentiles of Caspase 3

Liver cell suspension was prepared and centrifuged. The supernatant that formed after centrifugation was discarded, and the pellet was suspended in PBS. Caspase 3 marker was added then the tube was incubated. PBS/BSA was used to wash cells and centrifuged and the supernatant was discarded. Finally, liver cells were suspended and then fixed until acquired by flow cytometry [28].

### 2.9. Statistical Analysis

Data were analyzed using the Statistical Package of Social Science (SPSS) program for Windows (Standard version 21). The normality of data was first tested with one-sample Kolmogorov-Smirnov test. Continuous variables were presented as mean ± SD (standard deviation) for parametric data. ANOVA test was used to compare more than 2 means, and paired *t*-test was used to compare paired data. Pearson correlation was used to correlate continuous data. For all abovementioned statistical tests, the threshold of significance is fixed at 5% level (*P* value). Results were considered significant when the probability of error was less than 5% (*P* < 0.05).

## 3. Results

### 3.1. Effects of Nilotinib and Stem Cell Exosome Treatments on Liver Function Tests

The administration of CCl_4_ for 9 weeks caused severe liver damage as characterized by significant increase in the activity of serum ALT and a significant decrease in the concentration in serum ALB. A significant reduction in the activity of serum ALT and elevation of serum ALB was detected in the groups treated with Nilotinib and stem cell exosomes compared with the CCl_4_ group. The combination treatment of Nilotinib and stem cell exosomes showed a more significant reduction in the activity of serum ALT and elevation in serum ALB than the other treated groups. There was no significance in the serum ALT activity and ALB concentration between the CCl_4_ group and the free media group (Tables 1 and 2).

### 3.2. Effects of Nilotinib and Stem Cell Exosome Treatments on Hepatic Oxidative Stress Parameters

The administration of CCl_4_ caused a significant increase of hepatic MDA, NO, and NOS compared with the control group. Nilotinib and stem cell exosome treatments significantly decreased the hepatic NO and NOS compared with the CCl_4_ group. Stem cell exosome treatment slightly decreased the increased levels of hepatic MDA (not significant) compared with the CCl_4_ group, but the Nilotinib treatment caused a significant decrease on hepatic MDA compared with the CCl_4_ group. The combination treatment between Nilotinib and stem cell exosomes showed a more significant reduction in the hepatic MDA, NO, and NOS than the other treated groups. There are no significance in MDA, NO, and NOS between the CCl_4_ group and the free media group (Table 3). The administration of CCl_4_ caused a significant decrease in hepatic catalase, SOD, and GSH contents compared with the control group. Nilotinib and stem cell exosome treatments significantly increased the hepatic SOD and GSH compared with the CCl_4_ group. Stem cell exosome treatment slightly increased the levels of hepatic catalase (not significant) compared with the CCl_4_ group, but the Nilotinib treatment caused a significant increase on catalase compared with the CCl_4_ group. The combination treatment between Nilotinib and stem cell exosomes showed a more significant elevation in the hepatic catalase, SOD, and GSH than the other treated groups. There was no significance in catalase, SOD, and GSH between the CCl_4_ group and the free media group (Table 3).

### 3.3. Effects of Nilotinib and Stem Cell Exosome Treatments on Hepatic Apoptotic Markers

**Figure 1:**
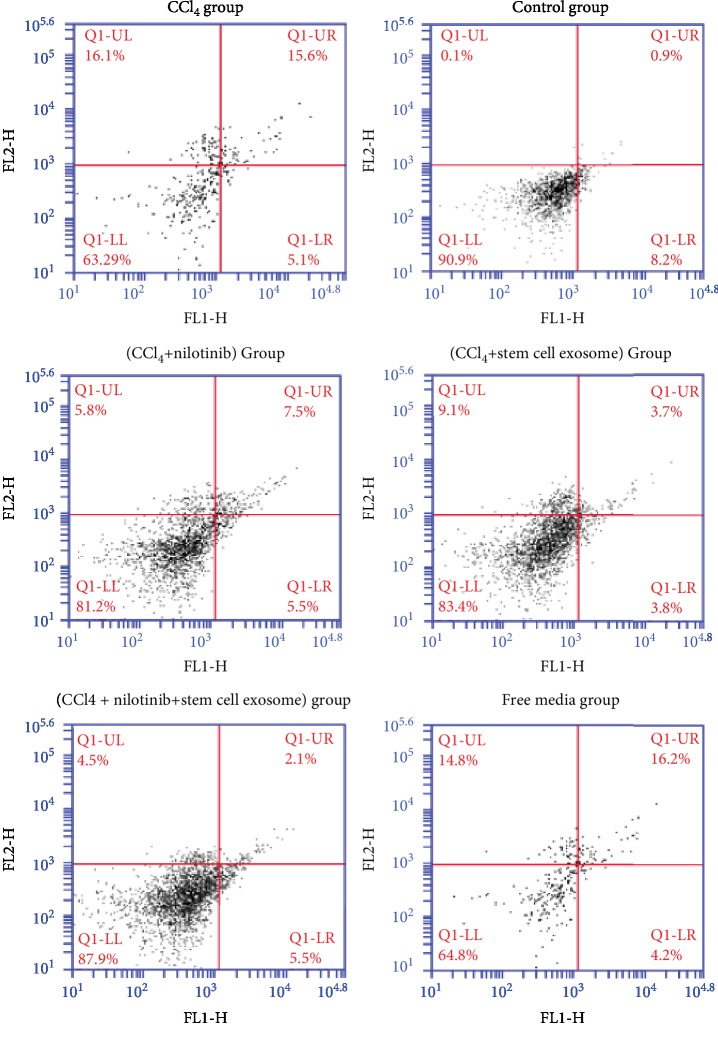
Annexin V apoptotic marker among the studied groups.

**Figure 2:**
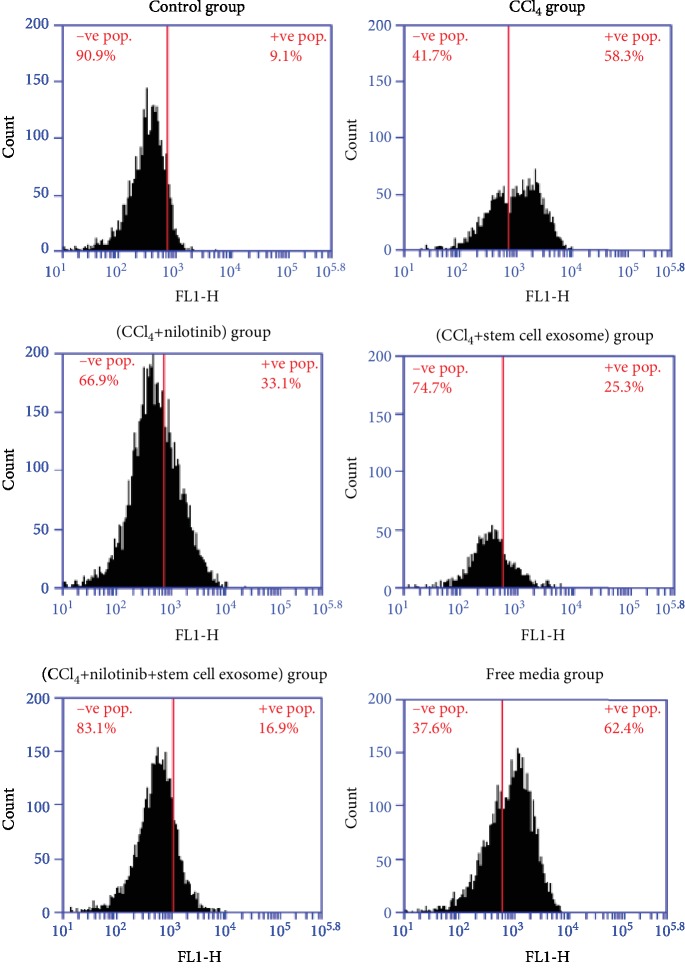
Caspase 3 apoptotic marker among the studied groups.

Annexin V marker showed that the administration of CCl_4_ caused a significant increase of apoptotic cells and necrotic cells' percentages compared with the control group and the viable cells were significantly decreased compared with the control group. Nilotinib and stem cell exosome treatments significantly decreased the apoptotic and necrotic cells and elevated the percentage of viable cells compared with the CCl_4_ group. The combination treatment between Nilotinib and stem cell exosomes showed a more significant reduction in the percentage of apoptotic and necrotic cells than the other treated groups. There was no significance between the CCl_4_ group and the free media group in the percentage of viable, necrotic, and apoptotic cells (Figure 1). The CCl_4_ administration caused a significant increase of caspase 3 percentage compared with the control group. Nilotinib and stem cell exosome treatments significantly decreased the percentage of caspase 3 compared with the CCl_4_ group. The combination treatment between Nilotinib and stem cell exosomes showed a more significant reduction in the percentage of caspase 3 than the other treated groups. There was no significance between the CCl_4_ group and the free media group in the percentage of caspase 3 (Figure 2).

### 3.4. Effects of Nilotinib and Stem Cell-Conditioned Medium Treatments on Hepatic Histopathology and Contents of 4-Hydroxyproline

**Figure 3:**
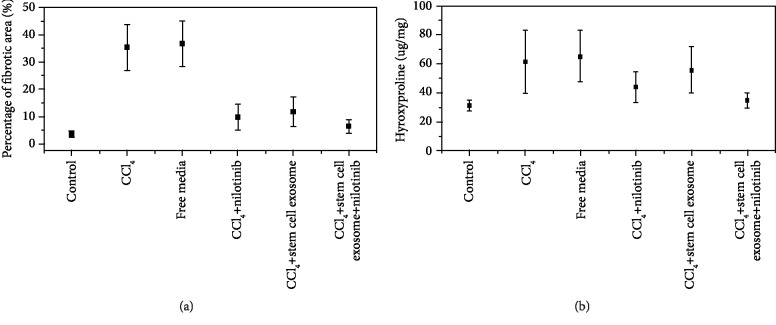
(a) Percentage of fibrotic area among the studied groups. (b) Hydroxyproline content among the studied groups.

**Figure 4:**
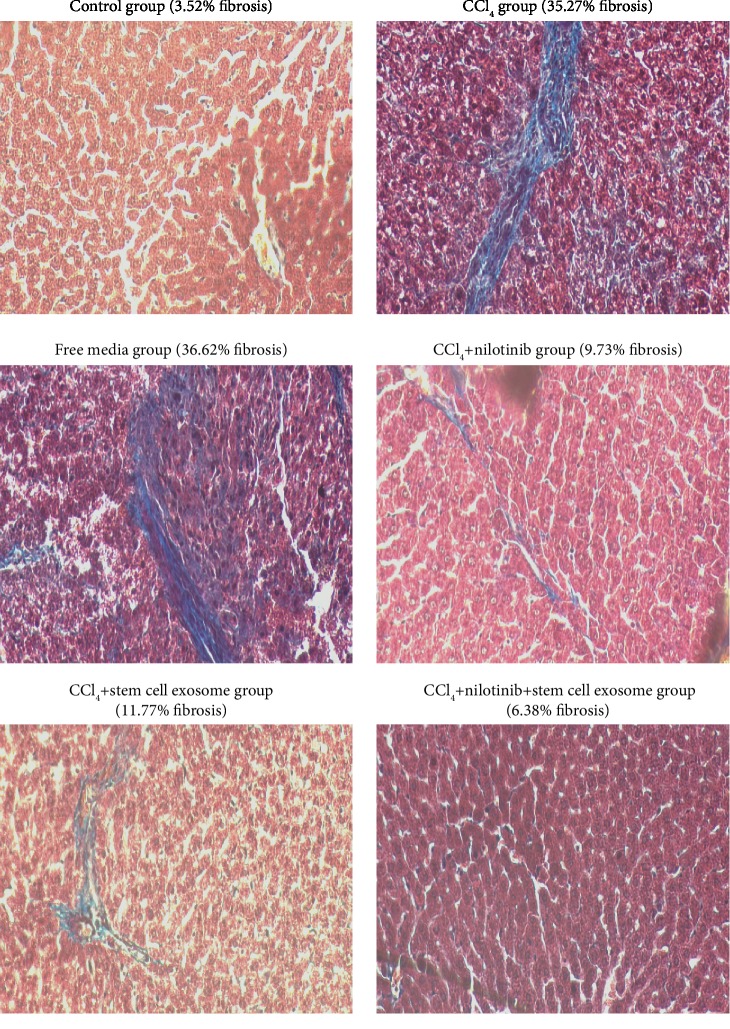
Histopathological examination among the studied groups.

The administration of CCl_4_ for 9 weeks caused marked inflammation, necrosis, and pronounced macrovesicular/microvesicular steatosis. According to Masson's trichrome-stained sections, the score of fibrosis was significantly elevated in rats treated with CCl_4_ compared with the control group. This increase was significantly decreased in rats treated with Nilotinib and stem cell exosomes. The combination treatment between Nilotinib and stem cell exosomes showed a more significant reduction in the fibrosis score than the other treated groups. There is no significance between the CCl_4_ group and the free media group in the fibrosis score (Figures 3 and 4).

CCl_4_ administration caused a significant increase of hepatic hydroxyproline compared with the control group. Nilotinib treatment significantly decreased the content of hydroxyproline compared with the CCl_4_ group, but the stem cell exosome treatment slightly decreased the content of hydroxyproline (not significant) compared with the CCl_4_ group. The combination treatment between Nilotinib and stem cell exosomes showed a more significant reduction in the hydroxyproline content than the other treated groups. There was no significance between the CCl_4_ group and the free media group in the levels of hydroxyproline (Figure 3).

## 4. Discussion

This study showed that the combination of Nilotinib and stem cell-conditioned media had significantly higher antifibrotic effect than each one alone. There were significant reductions of hydroxyproline content, fibrotic area, apoptotic markers, and oxidative stress in the combination group compared with each one alone. To the best of our knowledge, this may be the first report about the antifibrotic effect of this combination.

**Figure 5:**
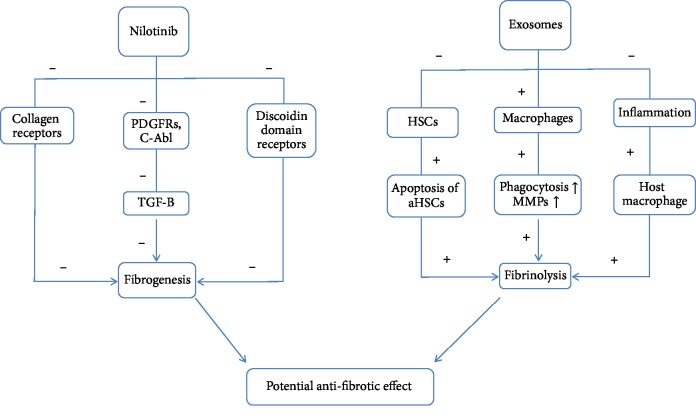
Potential synergetic antifibrotic effect of MSC-MC and Nilotinib combination.

The antifibrotic effect of the combination of Nilotinib and stem cell-conditioned media may be due to a synergistic effect of the two types of treatment (Figure 5). Mesenchymal stem cell-conditioned media have anti-inflammatory effects and reduce damages to hepatocytes which stop the activation of hepatic stellate cells (HSC) leading to apoptosis of HSC and fibrinolysis activation [11]. Alternatively, MSCs change the polarity of macrophages towards an anti-inflammatory phenotype, increase the production of matrix metalloproteinases to reduce the ECM, and increase the ability of phagocytosis of hepatocyte debris (during this process, macrophages increase the levels of proregenerative factors) [29]. On the other hand, Nilotinib acts as an antifibrotic agent through three major pathways involved in fibrogenesis: suppression of PDGFRS and c-Abl tyrosine kinase activities that induce TGF-*β* causing fibrogenesis, inhibition of collagen receptors, and downregulation of discoidin domain receptors [4], so MSC-CM leads to increase fibrinolysis and Nilotinib can decrease fibrinogenesis.

Concerning stem cell-conditioned media, Khajehahmadi et al. studied the effect of bone marrow-derived stem cell-conditioned medium (BMSCs-CM) in liver fibrosis which was induced by thioacetamide in male Wistar rats and concluded that the conditioned media had a positive anti-inflammatory and healing effect in rearrangement of hepatic fibrosis [30]. Moreover, they reported that the conditioned medium administration can overcome the immune reactivity, genomic instability, unexpected differentiation, and oncogenicity of stem cell transplantation [30].

Also, our results were in agreement with Chen et al., who studied the effect of MSC-CM cocultured with hepatocytes on acute liver failure (ALF) induced by D-galactosamine in rats, and then, results showed that mesenchymal stem cell-conditioned media, which integrates the therapeutic potentials of hepatocytes and stem cells, had superior performance at promoting the recovery of damaged hepatocytes and reversing D-galactosamine-induced ALF [31].

Regarding Nilotinib, our data were consistent with Qu et al. who reported that Nilotinib had potential for treating liver fibrosis with the least harmful effect compared to other tyrosine kinases [32], and these results are matched with our pervious data that proved Nilotinib was safer than Imatinib as an antifibrotic agent and this was evident in the reduction of liver hydroxyproline content, oxidative stress, and histopathology [4].

In our study, we further confirmed this antifibrotic effect by quantitative image analysis of liver tissue which is more effective than qualitative and semiquantitative methods [19]. Both Nilotinib and stem cell-conditioned media showed antifibrotic effect, but there was no significant difference between the Nilotinib and stem cell-conditioned medium treatment against CCl_4_-induced liver fibrosis in rats. However, there was a high significant difference between combination treatment and each one alone (*P* value < 0.001).

The strength of this work was the use of 2 different therapeutic modalities which proved to be a synergy effect and safe paving the way to its potential use in clinical trials. Also, the quantitative image analysis of fibrotic areas in our work added more evidence for proper evaluation of the antifibrotic effect of therapeutic agents and making the comparison more accurate.

The limitation of our work was that it is a single-center experience which needs validation in other centers. Also, we studied the effect of MSC-CM as a whole, while it is a mixture of different exosomes, and our work did not show the specific exosomes or which had this antifibrotic effect and did not show the specific active ingredient which is responsible for the antifibrotic effect.

## 5. Conclusion

Nilotinib combined with stem cell-conditioned media showed a synergistic effect and was a more effective antifibrotic than each one alone. The safety of both lines of the combined treatment may allow its use in clinical trials.

## Figures and Tables

**Table 1:** ALT ± SD (U/l) among the studied groups after 3 weeks, 6 weeks, and 9 weeks.

ALT (U/l)	ALT (U/l) (3 weeks)	ALT (U/l) (6 weeks)	ALT (U/l) (9 weeks)	Paired *t*-test		
				*P*_1_	*P*_2_	*P*_3_
Control	25.40 ± 3.3	26.70 ± 3.19	25.20 ± 3.01	*t*_1_ = 1.22 *P*_1_ = 0.25	*t*_2_ = 0.11 *P*_2_ = 0.91	*t*_3_ = 0.89 *P*_3_ = 0.39
CCl_4_	63.90 ± 20	82.30 ± 11.87	66.00 ± 18.92	*t*_1_ = 2.45 *P*_1_ = 0.04^∗^	*t*_2_ = 0.18 *P*_2_ = 0.86	*t*_3_ = 2.08 *P*_3_ = 0.06
Free media	62.30 ± 19.7	81.40 ± 13.78	64.60 ± 18.07	*t*_1_ = 2.05 *P*_1_ = 0.07	*t*_2_ = 0.29 *P*_2_ = 0.78	*t*_3_ = 2.22 *P*_3_ = 0.05^∗^
CCl_4_+Nilotinib	43.00 ± 14	51.70 ± 13.27	51.20 ± 11.22	*t*_1_ = 3.56 *P*_1_ = 0.006^∗^	*t*_2_ = 1.32 *P*_2_ = 0.22	*t*_3_ = 0.09 *P*_3_ = 0.93
CCl_4_+stem cell exosomes	55.90 ± 11.5	54.20 ± 10.42	38.30 ± 6.49	*t*_1_ = 0.33 *P*_1_ = 0.75	*t*_2_ = 4.39 *P*_2_ = 0.002^∗^	*t*_3_ = 4.77 *P*_3_ = 0.001^∗^
CCl_4_+stem cell exosomes+Nilotinib	30.50 ± 6.3	31.10 ± 7.70	29.00 ± 8.95	*t*_1_ = 0.19 *P*_1_ = 0.85	*t*_2_ = 0.46 *P*_2_ = 0.65	*t*_3_ = 0.99 *P*_3_ = 0.34
ANOVA test	13.87	37.81	19.78	—	—	—
*P* value	<0.001^∗^	<0.001^∗^	<0.001^∗^			

**Table 2:** ALB ± SD (U/l) among the studied groups after 3 weeks, 6 weeks, and 9 weeks.

Albumin	ALB (U/l) (3 weeks)	ALB (U/l) (6 weeks)	ALB (U/l) (9 weeks)	Paired *t*-test		
				*P*_1_	*P*_2_	*P*_3_
Control	4.07 ± 0.13	4.06 ± 0.09	4.08 ± 0.09	*t*_1_ = 0.25 *P*_1_ = 0.81	*t*_2_ = 0.43 *P*_2_ = 0.68	*t*_3_ = 0.56 *P*_3_ = 0.59
CCl_4_	3.86 ± 0.30	2.98 ± 0.49	2.42 ± 0.64	*t*_1_ = 4.36 *P*_1_ = 0.002^∗^	*t*_2_ = 6.02 *P*_2_ ≤ 0.001^∗^	*t*_3_ = 2.18 *P*_3_ = 0.06
Free media	3.80 ± 0.31	2.88 ± 0.50	2.47 ± 0.60	*t*_1_ = 4.27 *P*_1_ = 0.002^∗^	*t*_2_ = 4.84 *P*_2_ = 0.001^∗^	*t*_3_ = 1.93 *P*_3_ = 0.08
CCl_4_+Nilotinib	3.93 ± 0.20	3.48 ± 0.46	3.28 ± 0.69	*t*_1_ = 2.58 *P*_1_ = 0.03^∗^	*t*_2_ = 2.61 *P*_2_ = 0.028^∗^	*t*_3_ = 0.76 *P*_3_ = 0.46
CCl_4_+stem cell exosomes	3.95 ± 0.19	3.14 ± 0.53	3.07 ± 0.40	*t*_1_ = 4.38 *P*_1_ = 0.002^∗^	*t*_2_ = 7.17 *P*_2_ ≤ 0.001^∗^	*t*_3_ = 0.32 *P*_3_ = 0.75
CCl_4_+stem cell exosomes+Nilotinib	3.97 ± 0.14	3.69 ± 0.35	3.88 ± 0.36	*t*_1_ = 2.26 *P*_1_ = 0.05^∗^	*t*_2_ = 0.64 *P*_2_ = 0.54	*t*_3_ = 1.09 *P*_3_ = 0.30
ANOVA test	1.679	10.96	18.38	—	—	—
*P* value	0.155	<0.001^∗^	<0.001^∗^			

**Table 3:** Oxidative stress markers among the studied groups.

Groups	Malonaldehyde (mmol/g tissue)	Nitric oxide (*μ*mol/g tissue)	Glutathione reduced (*μ*mol/g protein)	Super oxide dismutase (U/mg protein)	Nitric oxide synthase (pmol/min/mg protein)	Catalase (mol/min/gm)
Control	77.93 ± 5.88	150.84 ± 19.25	23.47 ± 3.56	21.09 ± 2.86	18.03 ± 2.61	1.05 ± 0.11
CCl_4_	111.17 ± 18.21	205.51 ± 25.30	14.78 ± 3.02	14.11 ± 3.96	28.36 ± 5.01	0.66 ± 0.24
Free media	110.71 ± 16.80	205.76 ± 26.19	14.51 ± 3.45	14.37 ± 3.82	29.27 ± 3.26	0.64 ± 0.22
CCl_4_+Nilotinib	83.97 ± 14.78	176.39 ± 22.37	23.85 ± 5.08	19.70 ± 4.42	20.14 ± 5.66	0.99 ± 0.21
CCl_4_+stem cell exosomes	96.480 ± 28.41	173.25 ± 26.41	21.32 ± 3.37	18.33 ± 3.25	20.04 ± 2.85	0.80 ± 0.26
CCl_4_+stem cell exosomes+Nilotinib	80.10 ± 17.83	148.02 ± 14.02	23.28 ± 5.24	22.80 ± 3.82	18.98 ± 3.27	1.02 ± 0.21
*P* value	<0.001^∗^	<0.001^∗^	<0.001^∗^	<0.001^∗^	<0.001^∗^	<0.001^∗^

## Data Availability

The original data used to support the findings of this study are available from the corresponding author upon request.
